# China should emphasize understanding and standardized management in diabetic cognitive dysfunction

**DOI:** 10.3389/fendo.2023.1195962

**Published:** 2023-06-21

**Authors:** Qingqing Yin, Yan Gao, Xinyu Wang, Shangbin Li, Xunyao Hou, Wenkai Bi

**Affiliations:** ^1^ Key Laboratory of Endocrine Glucose & Lipids Metabolism and Brain Aging, Ministry of Education, Department of Geriatrics, Shandong Provincial Hospital Affiliated to Shandong First Medical University, Jinan, Shandong, China; ^2^ Department of Geriatric Neurology, Shandong Provincial Hospital Affiliated to Shandong First Medical University, Jinan, Shandong, China; ^3^ Department of Endocrinology, Shandong Provincial Hospital Affiliated to Shandong First Medical University, Jinan, Shandong, China

**Keywords:** diabetic cognitive dysfunction, dementia, understanding, standardized management, China

In recent years, the prevalence of diabetes has increased in China: the prevalence rate of diabetes in adults has reached 11.6% (1). In addition to causing macrovascular and microvascular lesions, diabetes also significantly affects the cognitive function of patients. Both diabetes and cognitive dysfunction are universally prevalent chronic diseases worldwide. Cognitive dysfunction affects many aspects of people with diabetes, including difficulty in self-glucose management, frequent episodes of severe hypoglycemia, and an increased risk of fractures, cardiovascular events, and death (2, 3). The importance of diabetic cognitive dysfunction recognition is explicitly mentioned in American Diabetes Association (ADA) 2021 guidelines, pointing to poor glycemic control being associated with cognitive dysfunction and longer duration of diabetes being associated with worse cognitive function (4). Therefore, it is necessary to strengthen the understanding and recognition of the cognitive dysfunction of diabetes mellitus. However, at present, the knowledge of diabetic cognitive dysfunction among physician internists in China is quite poor, and there is a lack of management and diagnosis guidance.

The risk of dementia in type 2 diabetes mellitus (T2DM) increases rapidly with age. The incidence of dementia in T2DM is 83/10000 in people aged 60 to 64 years and more than 1000/10000 in people over 85 years of age (5). Medical data from the veterans of the United States show that the prevalence of dementia in diabetes patients aged 65 to 74 years is 8.0%, and the prevalence of dementia in diabetes patients aged 75 years and older is as high as 18.3% (6). In a large meta-analysis of 24 longitudinal studies, the age-specific incidence of dementia ranged from 50/10000 for individuals aged 60 to 64 years and 600 to 800/10000 for those 85 years and older (7). T2DM increases the risk of dementia, but incidences of dementia among diabetics in China are relatively rarely reported. Therefore, China should pay more attention to the investigation of the incidence of dementia in diabetic patients.

Insulin resistance is the pathophysiological basis of T2DM and also a core mechanism of cognitive dysfunction in T2DM (2). Insulin degrading enzymes are simultaneously involved in insulin and β-amyloid (Aβ) degradation clearance. The insulin signaling pathway is impaired in the brain of T2DM, and the insulin level is increased, competing with Aβ, the degradation of the insulin degradation enzyme, and interfering with Aβ clearance. In addition, the insulin signaling transduction pathway can inhibit Aβ production and the hyperphosphorylation of microtubule-related protein tau through multiple pathways, so impaired insulin signaling can accelerate the progression of Alzheimer’s disease (AD) (8). Long-term chronic hyperglycemia affects neuron synapse formation, enhances oxidative stress response, leads to advanced glycation end products (AGEs) accumulation, accelerates atherosclerotic plaque formation, and causes neuron and vascular damage (2). Insulin resistance and neuroinflammation are interrelated pathological features, and they are considered as two principals responsible for synaptic disruption and neurophysiological changes, directly or indirectly (9). Brain insulin resistance occurs through the release of pro-inflammatory cytokines; peripherally produced pro-inflammatory cytokines such as TNF-α, IL-6, IL-12 and IL-1β can cross the blood-brain barrier, leading to neuroinflammation and central insulin resistance (10). Distorted lipid metabolism and vascular damage are also associated with the occurrence of cognitive dysfunction in T2DM patients. Distorted lipid metabolism increases oxidative stress by generating free radicals, causing cerebral small vessel disease and impaired cerebral perfusion (11). T2DM promotes atherosclerotic plaque formation, increasing the incidence of cerebrovascular events and leading to vascular dementia or mixed dementia (12). Cerebral white matter osteoporosis caused by demyelinating or brain microvascular diseases is common in AD patients and is also a risk factor of cognitive dysfunction in patients with T2DM (13). On the one hand, the damage to neurons caused by poorly controlled diabetes leads to amyloid and tau deposition in dementia. On the other hand, poorly controlled diabetes damages blood vessels in the brain, leading to vascular dementia. Diabetes is therefore not only a high risk factor for vascular dementia, but also an independent risk factor that increases the risk of AD (14, 15). Fully understanding and conducting early assessment and prevention of the above risk factors, and actively exploring new early diagnosis targets, can help to delay or avoid the occurrence of diabetic cognitive dysfunction, reduce the probability of long-term dementia, and improve the quality of life of patients.

Early recognition of cognitive dysfunction in diabetes patients is important. In patients with T2DM, risk factors such as anxiety and depression, repeated hypoglycemia, unexplained falls, and difficulty in self-glucose management are key monitoring targets for cognitive dysfunction. In the early stage, patients with diabetic cognitive dysfunction experience some overlooked clinical signs, such as changes in gait or eye movement responses, including decreased speed, longer stride time, increased stride variability, and decreased balance. It would be valuable to catch these useful indicators in time for routine screening in primary care. With the development of digital processes and medical artificial intelligence, clinically meaningful behavioral information is captured through the wearing of electronic devices to provide a basis for adjunctive diagnosis and risk assessment of cognitive dysfunction in diabetes (16).

A comprehensive assessment in the medical, functional, psychological, and social fields should be considered for elderly patients with diabetes to determine treatment goals and treatment options in order to help patients manage their diabetes. The content should include firstly an annual neuropsychological assessment screening for early identification of MCI or dementia in adults ≥65 years of age. Elderly patients with diabetes should be carefully screened and monitored for cognitive dysfunction, especially when dementia is suspected. Several simple neuropsychological assessment tools are available for screening cognitive disorders, such as the mini-mental status examination (MMSE) and the Montreal cognitive assessment scale (MoCA). Secondly, screening for cognitive dysfunction should be considered when patients experience a significant decline in clinical status due to problems with self-care activities (such as miscalculation of islet dose, difficulty in calculating carbohydrates, etc.) (17). Although glucagon-like peptide 1 receptor agonist (GLP-1RA) has been shown to improve cognitive and olfactory abnormalities in obese diabetic patients (18). However, olfactory dysfunction is not currently classified as part of neuropathy. In the future, it may be a risk indicator for predicting cognitive dysfunction in diabetes. Brain imaging changes related to cognitive function in patients with diabetes mainly include changes in brain parenchyma and cerebrovascular changes, remodeling of fiber connections, changes in activation degree of brain regions, changes in brain metabolism, etc. Imaging examination is one of the conditions for assessing cognitive dysfunction. MRI is a routine test for diagnosis and differential diagnosis of dementia; MRI may be helpful in identifying the prognosis of dementia (19). Functional imaging is not a routine diagnostic test for dementia, but SPECT and PET can be used to improve the accuracy of diagnosis with clinical suspicion. Cerebrospinal fluid T-tau, P-tau181, and Aβ were available for diabetic patients highly suspicious of AD after neuropsychological scale assessment (20). The detection of blood biomarkers for AD have promising application prospects, but currently blood biomarkers for AD have not been recognized and approved, so they are not recommended as routine tests for dementia and cognitive dysfunction (21).

Cognitive dysfunction has become one of the leading causes of death and disability in the older adults, and the risk of cognitive dysfunction is significantly increased in patients with diabetes. The high disability rate of cognitive dysfunction, the lost ability to live independently in the later stage, has brought a heavy economic burden and nursing burden onto families and society. Early identification and intervention of the risk factors of cognitive dysfunction, good blood glucose control, selection of appropriate hypoglycemic drugs, and routine treatment of cognitive dysfunction are the main methods for the treatment of diabetic cognitive dysfunction (22). Maintaining a good lifestyle is conducive to reducing the risk of diabetic cognitive dysfunction. Older adults that are in good health, with few chronic diseases and intact cognitive function, should set a lower target of blood glucose control (HbA_1c_ below 7.0%-7.5%). For patients with multiple chronic diseases and cognitive dysfunction, the goal of blood glucose control (HbA_1c_ below 8.0%-8.5%) should be more lenient (22). Different hypoglycemic drugs have different effects on cognitive function. Metformin and GLP-1RA may improve cognitive function; the effects of sulfonylureas, thiazolidinediones, dipeptidyl peptidase IV inhibitors, and sodium-glucose cotransporter 2 inhibitors on cognitive function are unclear (23, 24). The specific treatment for diabetic cognitive dysfunction is similar to that for other non-diabetic cognitive dysfunction patients. The process of dementia is Aβ accumulation and aggregation, promoting its clearance through specific anti-Aβ antibodies is a rational strategy, using monoclonal antibodies against amyloid tangle formation (25). However, until now, the results of the use of monoclonal antibodies have shown a lack of clinical efficacy and were not sufficient to meet the normal approval process. Compared to glycemic control and the choice of safer and more economical drugs to delay the onset of cognitive dysfunction, the choice of monoclonal antibodies for the long-term treatment of patients with diabetic cognitive dysfunction is a questionable issue (26). It is important to note that when using AD drugs, physicians should consider the overall condition of patients with diabetes rather than treating the cognitive dysfunction alone. Early detection of cognitive dysfunction is important for diabetes care. When clinicians treat patients with cognitive dysfunction, it is critical to simplify medication regimens, engage in supportive treatment, and enhance all aspects of patient care. For the daily care pathways of patients with diabetic cognitive dysfunction, cognitive training slows the progression of dementia to a certain extent. Compared to pharmacological interventions, cognitive training has no adverse effects and is a safe adjunctive treatment. Cognitive training can improve not only memory function, executive function, attention, language function, and overall cognitive function in patients with mild cognitive dysfunction but also depressive symptoms and other psychiatric symptoms (27).

In conclusion, there is currently no specific treatment option for patients with diabetes with MCI or dementia, and the treatment approach is consistent with that for patients with MCI alone or dementia. The treatment of patients with cognitive dysfunction should control risk factors (diabetes, hypertension, stroke, etc.), non-drug treatment (physical exercise, lifestyle intervention, cognitive training), drug treatment (folic acid, vitamin B12, etc.), and drug symptomatic treatment (ChEI, ergot alkaloids, glutamate receptor antagonists). Currently, there are no drugs recommended by the US Food and Drug Administration for the treatment of MCI symptoms, and the efficacy of drugs for the treatment of cognitive dysfunction remains to be further confirmed. Therefore, it is particularly important to actively prevent or delay the occurrence and development of dementia. Because of the slow progression of cognitive dysfunction in patients with diabetes, most patients with cognitive dysfunction have a good prognosis, especially those under 60 years of age. In clinical work, it is necessary to further strengthen the publicity of cognitive health care for the elderly and provide extensive education so as to carry out effective primary and secondary prevention.

## Author contributions

All authors contributed to the article and approved the submitted version.
